# Small activating RNA activation of ATOH1 promotes regeneration of human inner ear hair cells

**DOI:** 10.1080/21655979.2022.2045835

**Published:** 2022-03-04

**Authors:** Yong-Li Zhang, Moorim Kang, Jian-Cheng Wu, Meng-Yao Xie, Ruo-Yan Xue, Qi Tang, Hua Yang, Long-Cheng Li

**Affiliations:** a Department of Otolaryngology, Peking Union Medical College and Chinese Academy of Medical Sciences, Peking Union Medical College Hospital, Beijing, China; b Ractigen Therapeutics, Nantong, Jiangsu, China; c Institute of Reproductive Medicine, School of Medicine, Nantong University, Nantong, Jiangsu, China

**Keywords:** Small activating RNA, RNA activation, hair cell, ATOH1, hearing loss

## Abstract

The loss of inner ear hair cells leads to irreversible acoustic injury in mammals, and regeneration of inner ear hair cells to restore hearing loss is challenging. *ATOH1* is a key gene in the development and regeneration of hair cells. Small activating RNAs (saRNAs) can target a gene to specifically upregulate its expression. This study aimed to explore whether small activating RNAs could induce the differentiation of human adipose-derived mesenchymal stem cells into hair cell-like cells with a combination of growth factors *in vitro* and thus provide a new strategy for hair cell regeneration and the treatment of sensorineural hearing loss. Fifteen small activating RNAs targeting the human *ATOH1* gene were designed and screened in 293 T and human adipose-derived mesenchymal stem cells, and 3 of these candidates were found to be capable of effectively and stably activating *ATOH1* gene expression. The selected small activating RNAs were then transfected into hair cell progenitor cells, and hair cell markers were examined 10 days after transfection. After transfection of the selected small activating RNAs, the expression of the characteristic markers of inner ear hair cells, POU class 4 homeobox 3 (POU4F3) and myosin VIIA (MYO7A), was detected. Human adipose-derived mesenchymal stem cells have the potential to differentiate into human hair cell progenitor cells. *In vitro*, small activating RNAs were able to induce the differentiation of hair cell progenitor cells into hair cell-like cells. Therefore, RNA activation technology has the potential to provide a new strategy for the regeneration of hair cells.

## Graphical Abstract

**Figure:**
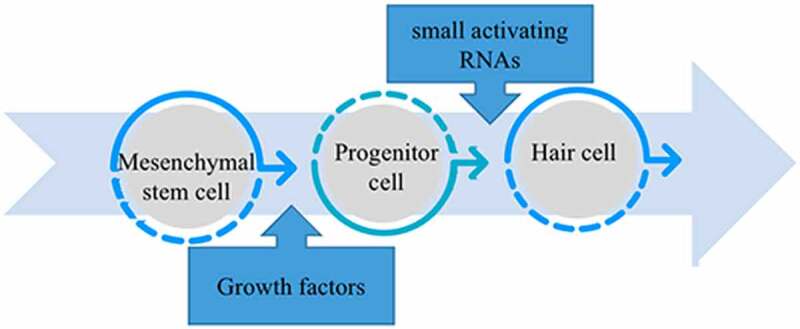

## Introduction

Sensorineural hearing loss is a common disease that affects human health. Damage or loss of cochlear hair cells is a common cause of sensorineural hearing loss. The hair cells of nonmammalian vertebrates, such as birds and zebrafish, can regenerate after injury. However, mammals lack the ability to spontaneously regenerate new hair cells. The regeneration of inner ear hair cells is a problem that has plagued human beings.

*ATOH1*, also referred to as *MATH1*, is a member of the basic helix-loop-helix (bHLH) transcription factor family and is a critical gene for the normal development and maintenance of inner ear hair cells. Studies have shown that the *ATOH1* gene plays an important role in the development of mammalian inner ear hair cells [^1–5^]. Overexpression of the *ATOH1* gene in vestibular supporting cells induces the regeneration of hair cells. Stem cells have both the ability to self-renew and differentiate and the potential to differentiate into hair cells after induction.

RNA activation (RNAa) is a mechanism mediated by small RNA molecules that leads to upregulated expression of specific genes. RNA activation is capable of promoting the expression of endogenous genes and is a novel technology that promotes the expression of specific genes in a targeted manner [^6–8^]. This study used RNA activation technology to induce the differentiation of human adipose-derived mesenchymal stem cells (MSCs) into hair cell-like cells and provides a new strategy for the regeneration of hair cells and the treatment of sensorineural hearing loss. The induced differentiation of stem cells and ATOH1 overexpression provide new methods for research into hair cell regeneration.

We assumed that small activating RNAs targeting the *ATOH1* gene have the potential of the hair cells regeneration. This study aimed to explore whether small activating RNAs targeting the *ATOH1* gene could induce the regeneration of inner ear hair cells *in vitro*. The goal of this research was to induce human adipose-derived mesenchymal stem cells into hair cells and find a new strategy for hair cells regeneration. In the present study, we found that small activating RNAs can induce the expression of the *ATOH1* gene. Human adipose-derived mesenchymal stem cells can be induced to gradually differentiate into hair cell-like cells under culture conditions through a combination of growth factors and small activating RNAs.

## Materials and methods

### Small activating RNA design

#### Small activating RNA (saRNA) design

The promoter sequence of human ATOH1 was retrieved from the UCSC Genome Database, and small activating RNA targets were selected as previously described [6]. The design rules include the following: (1) The sense DNA sequence of the promoter was used as the template. (2) The targeted promoter region was between −100 and −1000 bp upstream of the transcription start site (TSS). (3) The size of the small activating RNA should be 19 nt. (4) The small activating RNA should have a GC content of 40–60%. (5) The 3′ end should have lower thermodynamic stability than the 5′ end of the small activating RNA. (6) The 18th and 19th positions counted from the 5′ end of targets should be ‘A/T’s. (7) Five or more consecutive nucleotides and simple repeat sequences should not be taken into consideration. (8) Nucleotides 20–23 (flanking the 3′ end of a target) should preferably be ‘A/T’s. A total of 15 19-nt small activating RNAs with dTdT overhangs were designed and chemically synthesized on a Dr. Oligo 48 synthesizer (Biolytic Lab Performance, Inc.). A duplex RNA (dsCon) that lacked homology with any human sequences served as a duplex control. The sequences of all RNA duplexes are listed in Table 1.

**Table 1.** Sequences for dsRNA

Duplexes dsRNA	Sense	Antisense
dsCon	ACUACUGAGUGACAGUAGA[dT][dT]	UCUACUGUCACUCAGUAGU[dT][dT]
RAG9-i	GGGCUGAAGUGAAGGAGUU[dT][dT]	AACUCCUUCACUUCAGCCC[dT][dT]
RAG9-1	GCGGCCUUCUCUCUGCUUU[dT][dT]	AAAGCAGAGAGAAGGCCGC[dT][dT]
RAG9-2	UUCUCUCUGCUUUGAGUGG[dT][dT]	CCACUCAAAGCAGAGAGAA[dT][dT]
RAG9-3	CCUCCAUUGGCUGAGAAGA[dT][dT]	UCUUCUCAGCCAAUGGAGG[dT][dT]
RAG9-4	AGCUUGGGCACCAGCCGAG[dT][dT]	CUCGGCUGGUGCCCAAGCU[dT][dT]
RAG9-5	GAGCAGGCUUGGGAGUCCU[dT][dT]	AGGACUCCCAAGCCUGCUC[dT][dT]
RAG9-6	AGCAGGCUUGGGAGUCCUC[dT][dT]	GAGGACUCCCAAGCCUGCU[dT][dT]
RAG9-7	GGUCCAGAAUCGCCCAGAG[dT][dT]	CUCUGGGCGAUUCUGGACC[dT][dT]
RAG9-8	GCACAUCUGACCCGAGUCA[dT][dT]	UGACUCGGGUCAGAUGUGC[dT][dT]
RAG9-9	CCGCGGUCGUGCACAUCUG[dT][dT]	CAGAUGUGCACGACCGCGG[dT][dT]
RAG9-10	CGGGUCCAGAAUCGCCCAG[dT][dT]	CUGGGCGAUUCUGGACCCG[dT][dT]
RAG9-11	CACACAAGAACUUUUCUCG[dT][dT]	CGAGAAAAGUUCUUGUGUG[dT][dT]
RAG9-12	ACACCGGAGUCGAAUUACA[dT][dT]	UGUAAUUCGACUCCGGUGU[dT][dT]
RAG9-13	AACUUUUCUCGGGGUGUAA[dT][dT]	UUACACCCCGAGAAAAGUU[dT][dT]
RAG9-14	UUGGGAGUCCUCUGCACAC[dT][dT]	GUGUGCAGAGGACUCCCAA[dT][dT]
RAG9-15	UUGGGCACCAGCCGAGAGC[dT][dT]	GCUCUCGGCUGGUGCCCAA[dT][dT]

## Cell culture and screening of small activating RNAs

The human embryonic kidney cell line 293 T (ATCC® CRL) and human adipose-derived mesenchymal stem cells (ScienCell) were used as the cell lines to screen the small activating RNAs. 293 T cells were cultured in Dulbecco’s modified Eagle’s medium (DMEM; Thermo Fisher) supplemented with 10% fetal bovine serum (FBS; Thermo Fisher). Mesenchymal stem cells were cultured in mesenchymal stem cell medium (MSCM, STEMCELL). Both cell types were cultured at 37°C in a 5% CO_2_ environment. The 293 T cells and mesenchymal stem cells were transfected with small activating RNAs at a concentration of 10 nM for 72 h, unless otherwise stated, using Lipofectamine™ RNAiMAX (Invitrogen) in accordance with the manufacturer’s instructions. Each experiment was repeated at least 3 times. For dose response experiments, cells were transfected with small activating RNAs at concentrations ranging from 1 nM to 100 nM.

## Induction of hair cell progenitor cells

Mesenchymal stem cells (passages 3–4) were used to generate inner ear hair cell progenitor cells. Specifically, mesenchymal stem cells were seeded into 6-well plates (Corning) at a density of 2 × 10^5^ cells per well. A mixed medium consisting of serum-free DMEM/F12 (Thermo Fisher) and N2/B27 (Thermo Fisher) was used as the basal induction medium for mesenchymal stem cells. To induce hair cell progenitor cells, we used the same induction conditions used by Jone et al. [9]. Mesenchymal stem cells were first cultured in serum-free medium containing epidermal growth factor (EGF) (20 ng/ml, STEMCELL) and insulin-like growth factor-1 (IGF-1) (50 ng/ml, STEMCELL) for 2 weeks and then cultured in serum-free medium containing basic fibroblast growth factor (bFGF) (20 ng/ml, Abcam) for 2 weeks. The medium was replaced with fresh medium once every 2 days for 4 weeks. As a control, mesenchymal stem cells were also cultured in serum-free basal induction medium for 4 weeks in the absence of EGF, IGF-1 and bFGF. The experiment was repeated at least 3 times.

## Induction of hair cell-like cells

The induced hair cell progenitor cells were maintained in 6-well plates (2 × 10^5^ cells per well) and in a mixed medium consisting of serum-free DMEM/F12 and N2/B27 and were transfected with ATOH1 small activating RNAs using RNAiMAX or an *ATOH1* plasmid (ORIGENE, #RC210192) using Lipofectamine 3000 for 10 days. Human pluripotent stem cells transfected with dsCon or transfected without duplex RNA (mock transfection) served as controls. The culture medium was changed every other day. The experiment was repeated at least 3 times.

## Real-time reverse transcription-quantitative polymerase chain reaction (RT–qPCR) and semiquantitative RT–PCR

Total RNA was isolated from treated cells using the RNeasy Plus Mini kit (Qiagen) in accordance with the manufacturer’s instructions. One microgram of the total RNA was reverse transcribed into complementary DNA (cDNA) using the PrimeScript RT reagent kit with gDNA Eraser (Takara) and the PrimeScript™ 1^st^ Strand cDNA Synthesis Kit (Takara). The obtained cDNA was subjected to qPCR using SYBR Premix Ex Taq II (Tli RNase H Plus) (Takara). The reaction conditions were as follows: 1 cycle of initial denaturation at 95°C for 30 sec; 40 cycles of PCR at 95°C for 5 sec and 60°C for 30 sec; 1 cycle of melting at 95°C for 5 sec and 60°C for 60 sec; 40 cycles of denaturation at 95°C for 5 sec and annealing/extension at 60°C for 30 sec; and 1 cycle of cooling to 50°C for 30 sec. The *HPRT* gene served as an internal control. The condition was constant for each primer, and each experiment was repeated at least 3 times. In addition, semiquantitative RT–PCR of the obtained cDNA was performed using hot-start enzyme (Takara), dNTPs (Takara) and specific primers. The reaction conditions were as follows: denaturation at 95°C for 30 sec; annealing at 60°C for 30 sec; extension at 72°C for 60 sec; 25–35 cycles. The *HPRT* gene served as an internal control. The experiment was repeated at least 3 times. The primers used for RT–qPCR and semiquantitative RT–PCR are listed in Table 2.

**Table 2.** Sequences of PCR primers

Gene	Forward	Reverse	Product size, bp
RT-qPCR primer			
ATOH1	CCCTTCCAGCAAACAGGTGA	GAACGACGGGATAACATTGCG	127
HPRT1	AAAGATGGTCAAGGTCGCAAG	TAGTCAAGGGCATATCCTACAAC	120
Semi-quantitative RT-PCR primer			
ATOH1	GCCGCCCAGTATTTGCTACA	GCTAGCCGTCTCTGCTTCTG	260
PAX2	GAGCGAGTTCTCCGGCAAC	GTCAGACGGGGACGATGTG	209
SOX2	ATGCACCGCTACGACGTGA	CTTTTGCACCCCTCCCATTT	437
PAX8	CAAGGTGGTGGAGAAGATTGG	CGGATGATTCTATTAATGGAGCTG	141
POU4F3	TGCAAGAACCCAAATTCTCC	GAGCTCTGGCTTGCTGTTCT	758
MYO7A	CACATCTTTGCCATTGCTGAC	AGAAGAGAACCTCACAGGCAT	644
GAPDH	GTTCGTCATGGGTGTGAACCA	AGTCCTTCCACGATACCAAAGT	132

## Western blotting assay

Cells were lysed with CST RIPA buffer and 0.1% SDS, and proteins were collected from the lysed cells using NE-PER extraction reagent. The protein concentration was determined using a bicinchoninic acid (BCA) protein assay (Thermo Fisher Scientific). The proteins were separated by electrophoresis on a 10% sodium dodecyl sulfate (SDS)-polyacrylamide gel and then electroblotted onto polyvinylidene fluoride membranes (GE Healthcare). The membranes were blocked with blocking solution (3% nonfat dry milk, Bio–Rad, #170-6404) for 2 h at room temperature, incubated with a recombinant anti-MATH1/HATH1 antibody (Abcam, #ab168374; 1:1000 dilution) and an anti-Tubulin antibody (Cell Signaling Tech, #2148s; 1:5000 dilution) as the primary antibodies at 4°C overnight and then incubated with secondary anti-rabbit antibodies (Cell Signaling Tech, #7074s; 1:10,000 dilution) at room temperature for 2 h. The results were imaged and recorded using a multifunctional gel imaging system (Bio–Rad, ChemiDoc^TM^ MP).

## Immunocytofluorescence assay

Treated cells were fixed in 4% paraformaldehyde for 10 min and then blocked with a blocking solution (10% normal donkey serum with 0.3% Triton X-100 in PBS) for 1 h at room temperature. Subsequently, the hair cell progenitor cells were characterized using an anti-PAX8 antibody (Abcam, #ab53490; 1:20 dilution), while the hair cell-like cells were characterized using an anti-myosin VIIa/MYO7A antibody (Abcam, #ab150386; 1:100 dilution) at 4°C overnight and then incubated with a secondary FITC goat anti-mouse Ig antibody (BD, #554,001) at room temperature for 1 h followed by counterstaining with DAPI.

## Results

In this study, we explored the potential of small activating RNAs to induce the differentiation of human hair cell cells *in vitro* and found a new strategy for hair cell regeneration. Our data showed that small activating RNAs can target the *ATOH1* gene and induce its expression. A combination of growth factors and small activating RNAs can induce human adipose-derived mesenchymal stem cells into hair cells step by step *in vitro.*

## Identification of ATOH1 small activating RNAs

**Figure 1.**
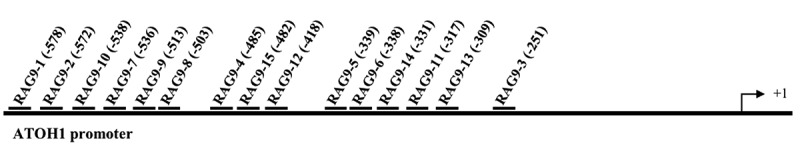
**Design of ATOH1 small activating RNAs**. The names of each small activating RNA target site relative to the transcription starting site (+1) on the ATOH1 promoter are indicated.

To induce ATOH1 gene expression, 15 small activating RNAs were designed to target the promoter region of the human *ATOH1* gene, ranging from position – 578 to 251 relative to the transcription start site (Figure 1).

**Figure 2.**
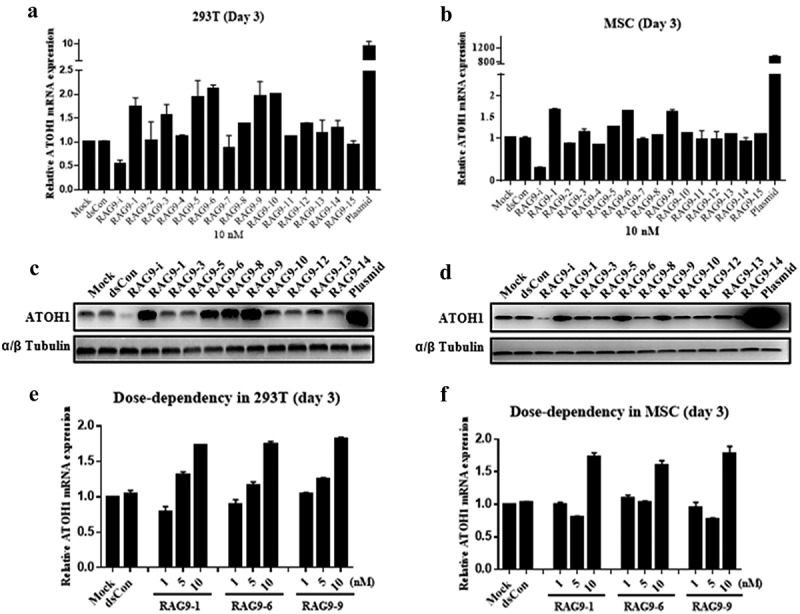
**Screening of ATOH1 small activating RNAs in 293 T cells and mesenchymal stem cells**. Cells were transfected with the indicated small activating RNAs using RNAiMAX at the indicated concentrations for 72 h. Mock samples were transfected in the absence of duplex RNA. Plasmid DNA was transfected at 1 µg/µl using Lipofectamine 3000. The transfected cells were subjected to RT–qPCR after RNA isolation, an RT reaction to generate cDNA and a Western blotting assay after protein isolation. A and B. Relative mRNA expression levels of the ATOH1 gene in 293 T cells (a) and mesenchymal stem cells (b) 72 h after transfection. C and D. ATOH1 protein levels assessed by Western blotting in 293 T cells (c) and mesenchymal stem cells (d) at 72 h after transfection. E and F. Relative ATOH1 mRNA expression levels in response to different small activating RNA concentrations.

As a control, an siRNA targeting ATOH1 mRNA (RAG9-i) was also designed. These small activating RNAs and the siRNA (RAG9-i) were transfected into 293 T and mesenchymal stem cells at 10 nM for 72 h, and the mRNA and protein expression of the *ATOH1* gene was examined by RT–PCR and Western blotting. As shown in Figure 2, RAG9-i transfection resulted in the expected downregulation of ATOH1 mRNA expression in both cell types, while ATOH1-expressing plasmid transfection resulted in a 9.07-fold and 945.8-fold increase in ATOH1 mRNA in 293 T cells and mesenchymal stem cells, respectively. Compared with mock and dsCon transfection, 6 of the 15 small activating RNAs (RAG9-1, RAG9-3, RAG9-5, RAG9-6, RAG9-9 and RAG9-10) produced a 1.5-fold or more increase in the expression of ATOH1 mRNA in 293 T cells, and 3 small activating RNAs (RAG9-1, RAG9-6 and RAG9-9) consistently upregulated the mRNA expression of the *ATOH1* gene in mesenchymal stem cells (Figure 2(a, b)). Induction of ATOH1 expression by these small activating RNAs could be readily verified at the protein level as assessed by Western blotting (Figure 2(c, d)). Therefore, these 3 small activating RNAs were selected as candidate small activating RNAs for further testing.

A dose–response transfection experiment demonstrated that a small activating RNA transfection concentration of 10 nM exhibited the highest potency in inducing ATOH1 mRNA expression for all 3 candidate small activating RNAs in both cell types (Figure 2(e,f)).

## Induction of hair cell progenitor cells

Our goal was to induce human adipose-derived mesenchymal stem cells into hair cells. We first transfected the 3 selected small activating RNAs into mesenchymal stem cells 3 times at a concentration of 10 nM. At 4 weeks after transfection, the expression levels of hair cell progenitor cell markers, such as paired box 2 (PAX2), SRY-box 2 (SOX2), and paired box 8 (PAX8), were evaluated. The expression of these 3 markers and that of the hair cell markers POU class 4 homeobox 3 (POU4F3) and myosin VIIA (MYO7A) was not detected (data not shown). Therefore, we concluded that small activating RNAs alone might not be able to directly induce mesenchymal stem cells into hair cell progenitor cells or hair cells. We therefore decided to first induce hair cell progenitor cells from mesenchymal stem cells and then examine whether small activating RNAs could induce hair cell progenitor cells into hair cells.

**Figure 3.**
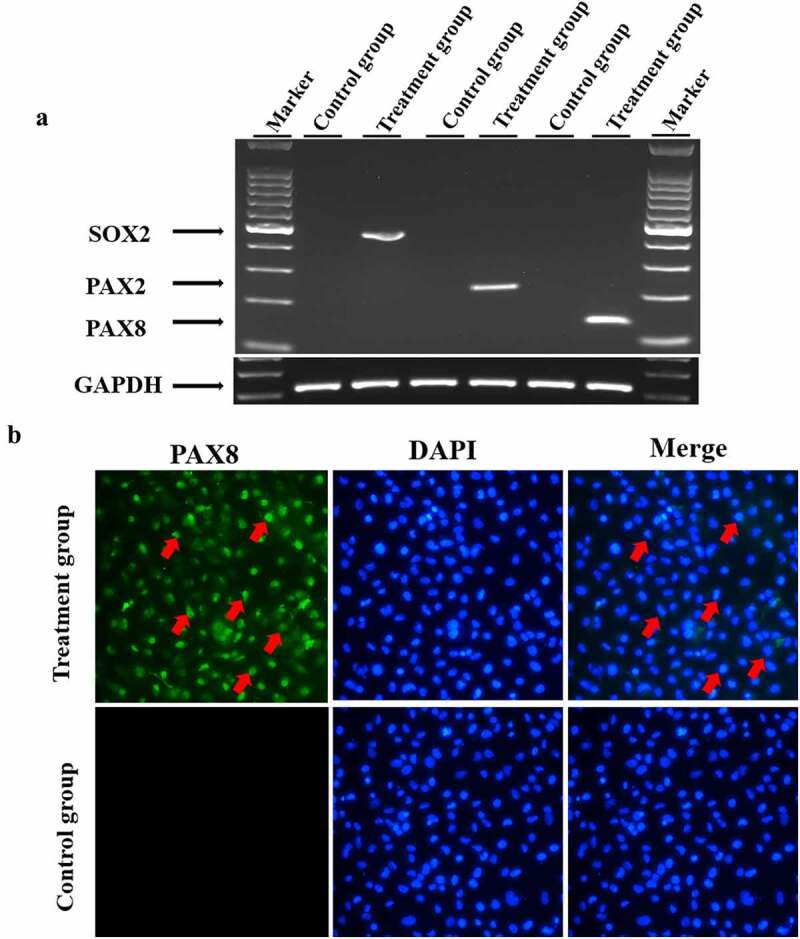
**Induction of hair cell progenitor cells**. Mesenchymal stem cells were cultured in induction medium containing EGF, IGF-1 and bFGF for 4 weeks, and hair cell progenitor cell marker genes were detected. **A**. Mesenchymal stem cells were cultured in hair cell progenitor cell induction medium as described in the Materials and Methods. Total cellular RNA was isolated from the treated cells and reverse transcribed into cDNA, which was amplified by semiquantitative RT–PCR. The *GAPDH* gene was also amplified as a control for RNA loading. B. Treated cells were fixed and stained with an antibody against PAX8, and the nuclei were counterstained. Red arrows denote positively stained cells.

To induce hair cell progenitor cells, mesenchymal stem cells were cultured in MSCM (ScienCell, #7501) for 3–5 passages. Afterward, the medium was replaced with serum-free hair cell progenitor cell induction medium containing EGF, IGF-1 and bFGF. Four weeks later, the mRNA expression of hair cell progenitor cell marker genes, including PAX2, SOX2 and PAX8, in the treated cells was detected by semiquantitative PCR. As shown in Figure 3(a), the expression of PAX2, SOX2 and PAX8 was induced in all treated cells, whereas no detectable expression was observed in untreated control cells. In addition, we detected the expression of PAX8 using immunofluorescence staining. Consistent with the RT–PCR results in Figure 3(a), treated cells showed positive staining for PAX8 compared to negative staining in untreated cells (Figure 3(b)). These results demonstrated that hair cell progenitor cells had been successfully induced.

## Induction of hair cell-like cells from hair cell progenitor cells by small activating RNAs

**Figure 4.**
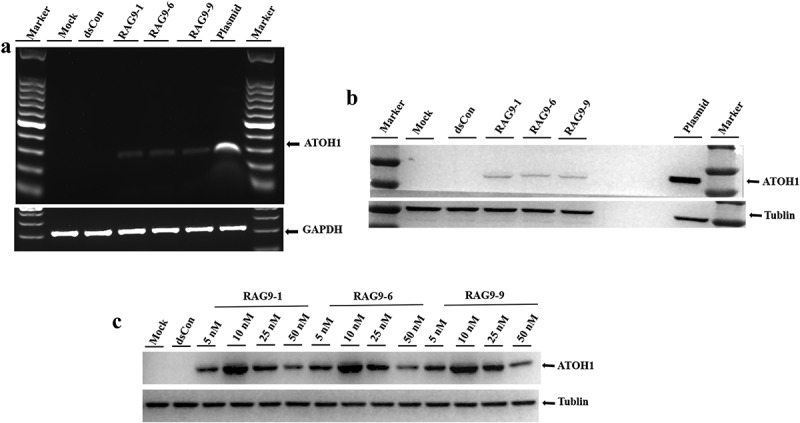
**Validation of selected small activating RNAs (RAG9-1, RAG9-6 and RAG9-9) in hair cell progenitor cells**. The 3 selected small activating RNAs, the plasmids containing the *ATOH1* gene and the dsRNA were transfected into hair cell progenitor cells to verify the activation effect of small activating RNAs in hair cell progenitor cells. Then, a dose–response transfection was performed to determine the most potent concentration. As shown in A and B, expression of the *ATOH1* gene was detected in the small activating RNA treatment group and the plasmid treatment group but not in the blank control group and the dsCon group. The results indicate that RAG9-1, RAG9-6 and RAG9-9 were able to activate the expression of the *ATOH1* gene in progenitor cells. As shown in C, the expression of the *ATOH1* gene was highest when the concentration of the 3 small activating RNAs was 10 nM. This result indicates that the most effective activation concentration for the small activating RNAs was 10 nM in hair cell progenitor cells.

To verify the activating effect of the small activating RNAs that were screened out of 293 T cells and mesenchymal stem cells on the expression of the *ATOH1* gene in inner ear hair cell progenitor cells and to determine whether the small activating RNAs were able to induce hair cell progenitor cells into hair cells, we first transfected the 3 selected small activating RNAs into hair cell progenitor cells. RT–PCR and Western blotting were then performed to examine the expression of the *ATOH1* gene in hair cell progenitor cells, and the effective activation concentration of these 3 small activating RNAs in hair cell progenitor cells was explored. The hair cell progenitor cells induced above exhibited no detectable expression of ATOH1 by either RT–PCR or Western blotting, while transfection of the ATOH1 plasmid induced strong ATOH1 mRNA and protein expression (Figure 4(a, b)). Hair cell progenitor cells transfected with all 3 candidate small activating RNAs exhibited obvious ATOH1 mRNA and protein expression (Figure 4(a, b)). A dose–response transfection in hair cell progenitor cells confirmed that all concentrations of small activating RNAs ranging from 5 nM to 50 nM induced ATOH1 protein expression, with 10 nM being the most potent concentration (Figure 4(c)), which is consistent with the dose–response results obtained from 293 T and mesenchymal stem cells (Figure 2(e, f)). It should be noted that the Western blot in Figure 4(b) was exposed for only 5 sec because of the extremely strong signal emitted by the plasmid-transfected sample, whereas the exposure time in Figure 4(c) was 30 sec. Therefore, all 3 selected small activating RNAs could be used to study the induction of inner ear hair cells, and their optimal activation concentration was 10 nM.

**Figure 5.**
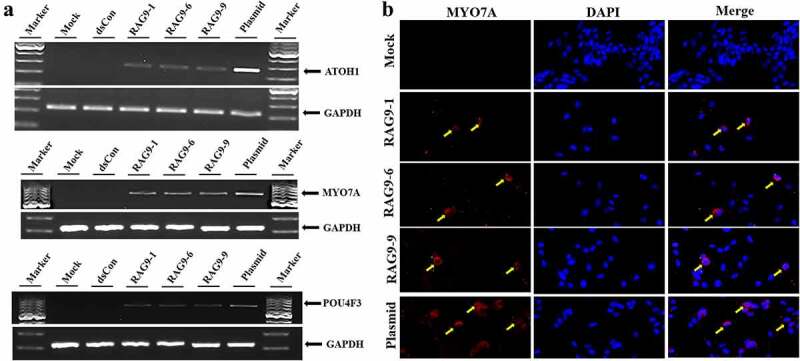
**Induction of hair cell-like cells**. Inner ear progenitor cells were transfected with the 3 selected small activating RNAs, the plasmid containing the *ATOH1* gene and dsRNA for 10 days, and hair cell markers (ATOH1, POU4F3 and MYO7A) were detected. A. The expression of hair cell markers (ATOH1, MYO7A, POU4F3 and GAPDH) was detected in the RAG9-1, RAG9-6, RAG9-9 and plasmid groups using semiquantitative RT–PCR. B. The expression of MYO7A was examined using immunofluorescence technology. The expression of MYO7A was detected in the RAG9-1, RAG9-6, RAG9-9 and plasmid groups (as shown by the yellow arrow).

To further verify whether small activating RNAs can induce the differentiation of inner ear progenitor cells into hair cells, we cultured inner ear progenitor cells in serum-free induction medium. We then transfected the 3 selected small activating RNAs, the plasmid containing the *ATOH1* gene and the dsRNA into the progenitor cells according to the grouping. At 10 days after transfection, we detected the expression of hair cell markers (ATOH1, POU4F3 and MYO7A) in the plasmid treatment group and the small activating RNA treatment group using PCR. In contrast, PCR failed to detect the expression of the above markers in the blank control group and the dsCon group (Figure 5(a)). In addition, we examined the expression of MYO7A in cells using immunofluorescence technology. We detected MYO7A expression in the plasmid treatment group and the small activating RNA treatment group but not in the blank control group or the dsCon group (Figure 5(b)).

## Discussion

The inner ear hair cells of birds and lower vertebrates can regenerate after damage, but those of mammals cannot. Stem cells are multifunctional cells with self-renewal and multiple differentiation potential. Studies have found that stem cells can be induced to differentiate into the progenitor cells of inner ear hair cells or into hair cells under certain *in vitro* conditions [^9–13^]. It has been reported that embryonic stem cells can be induced to differentiate into inner ear progenitor cells *in vitro* under the action of cytokines such as EGF, IGF-1 and bFGF [9,10]. Oshima et al. found that the inhibition of Wnt and transforming growth factor-beta (TGF-β) and activation of the IGF1 signaling pathway in induced pluripotent stem (iPS) cells induce the formation of ectoderm [13]. Subsequently, bFGF induces ectodermal cells to differentiate into inner ear progenitor cells, which eventually differentiate into hair cells under growth factor-free adherent culture conditions. All these studies have confirmed that stem cells have the potential to differentiate into hair cell progenitor cells or hair cells under suitable conditions [^9–13^]. In these studies, we used induction conditions similar to those used in previous studies to induce human adipose-derived mesenchymal stem cells into inner ear progenitor cells [9]. Inner ear progenitor cells were induced after human adipose-derived mesenchymal stem cells were cultured under the induction condition of growth factors, including EGF, IGF-1 and bFGF. Our study obtained results similar to those of previous studies [^9–13^] and confirmed that human adipose-derived mesenchymal stem cells can also differentiate into hair progenitor cells.

As a member of the bHLH family, ATOH1 is an indispensable transcription factor in the development of inner ear hair cells [^14–20^]. Bermingham et al. failed to detect the presence of hair cells in mouse inner ears after the *ATOH1* gene was knocked out, whereas overexpression of the *ATOH1* gene allowed the detection of hair cells in cochleae cultured *in vitro* [14]. This study has shown that the *ATOH1* gene is very important for the development of inner ear hair cells. Zheng et al. found that the overexpression of ATOH1 led to the generation of ectopic hair cells in rat cochleae cultured *in vitro*, and a specific marker of hair cells (Myosin7a) was detected in these ectopic cells [15]. Later, Kawamoto et al. found that overexpression of ATOH1 in Guinea pig cochleae achieved the same effect [16]. Zheng et al and Kawamoto et al confirmed that overexpression of the *ATOH1* gene can lead to hair cells in some conditions [15,16]. However, in their studies, they used the *Atoh1* plasmid or *Atoh1* cDNA to induce hair cells in rat or mature Guinea pigs [15,16]. There are no studies that use small activating RNAs to induce hair cell regeneration in human cells. In our study, we transfected small activating RNA into human inner ear progenitor cells and obtained hair cells. Our study found that overexpression of the *ATOH1* gene with small activating RNAs can lead to hair cells in human cells and confirmed that the *ATOH1* gene plays an important regulatory role in the process of hair cell regeneration.

Small activating RNAs are short duplex RNAs that target promoter sequences of a gene to specifically upregulate its expression via a mechanism known as RNA activation [^6–8,^^21^]. Researchers have used small activating RNAs to induce the expression of a variety of genes in cultured cells and *in vivo* [^22–28^]. The first clinical stage small activating RNA, MLT-CEBPA, has been shown to be safe and well tolerated for human use and has demonstrated efficacy in treating liver cancer [22,26]. Another application of small activating RNAs is to induce cell reprogramming by activating specific genes. In this regard, Wang et al. used small activating RNAs to activate OCT4 in human adipose-derived stem cells and achieved partial reprogramming of iPS cells by replacing the OCT4 vector with small activating RNAs [27]. Activation of NANOG by its small activating RNAs has also been shown to maintain embryonic carcinoma cells in an undifferentiated state in the presence of retinoic acid [28]. Other studies have shown that other kinds of RNA, such as microRNAs and long noncoding RNAs, can also promote mesenchymal stem cell differentiation [29,30], which shows that multiple differentiation potentials of mesenchymal stem cells can be promoted by different RNAs. Therefore, targeted activation of the ATOH1 gene by small activating RNAs represents an attractive approach in regenerating hair cells from other cell types, especially mesenchymal stem cells.

In this study, we used a method that combined growth factors (EGF, IGF-1 and bFGF) and small activating RNAs to gradually induce mesenchymal stem cells into hair cell-like cells. First, we used growth factors to induce mesenchymal stem cells into inner ear progenitor cells, and the inner ear progenitor cell induction method was similar to that used by Jone et al. [9]. Then, the selected small activating RNAs were transfected into hair cell progenitor cells. At the end of the hair cell induction period, we detected the expression of the hair cell markers ATOH1, POU4F3 and MYO7A. We did not investigate the morphology, structure or electrophysiological function of induced cells in terms of the characteristic markers of hair cells. Therefore, these cells were called hair cell-like cells. In this study, we first used small activating RNAs to induce human adipose-derived mesenchymal stem cells. However, no hair cell markers were detected after human adipose-derived mesenchymal stem cells were transfected with small activating RNA only. We speculated that activation of *ATOH1* gene expression alone in human adipose-derived mesenchymal stem cells was not enough to promote the differentiation of human adipose-derived mesenchymal stem cells into hair cells. However, it is unclear whether small activating RNAs targeting the *ATOH1* gene can induce inner ear progenitor cells into hair cells. Therefore, we carried out further verification by establishing a small activating RNA treatment group, a plasmid treatment group, a blank control group and a dsCon group. At the end of the hair cell induction period, we detected the expression of the hair cell markers ATOH1, POU4F3 and MYO7A in the plasmid treatment group and the small activating RNA treatment group. In contrast, the expression of these hair cell markers was not detectable in the blank control group or the dsCon group. It can be concluded that growth factors alone were not sufficient to induce the differentiation of human adipose-derived mesenchymal stem cells into hair cells. Only the combination of growth factors and small activating RNAs or the plasmid-mediated overexpression of the *ATOH1* gene could gradually induce the change of human adipose-derived mesenchymal stem cells into hair cells. Unlike previous techniques, such as plasmid and gene editing, RNA activation technology achieves target gene overexpression by activating the expression of endogenous genes. This process works at the transcriptional and epigenetic levels. It does not alter the genome while activating the expression of the target genes, which largely retains the integrity of the endogenous genes and the natural structure of the target gene mRNA. RNA activation technology has many target genes that could provide new solutions to the lack of suitable targets for traditional targeted therapies (such as antibodies and small molecules). In addition, small activating RNAs have the advantages of a small molecular weight, multiple targets and ease of chemical synthesis. Therefore, small activating RNAs have great application and transformation potential. To date, there is a lack of studies related to the application of RNA activation technology in the regeneration of inner ear hair cells. The results of our study showed that the combination of small activating RNAs and growth factors was able to induce the differentiation of human adipose-derived mesenchymal stem cells into hair cell-like cells, indicating that RNA activation technology has great potential in the study of inner ear hair cell regeneration. This was an *in vitro* study focusing on the application of RNA activation technology for hair cell regeneration. We have not yet conducted any animal research using RNA activation technology. The safety and effectiveness of RNA activation technology when used to induce inner ear hair cell regeneration in animals is not yet clear, nor is the ability of animals to tolerate RNA activation. Additional related research is necessary.

## Conclusion

In the present study, ATOH1 small activating RNAs that robustly activated ATOH1 mRNA and protein expression in different cells were designed and identified, and small activating RNA treatment was used to induce hair cell progenitor cells into hair cell-like cells. The results of this study provide a rationale for further testing of small activating RNAs in more relevant model systems to determine their clinical utility in regenerating hair cells to restore hearing loss.
